# The Effects of Systemic and Local Acidosis on Insulin Resistance and Signaling

**DOI:** 10.3390/ijms20010126

**Published:** 2018-12-30

**Authors:** Nicola Baldini, Sofia Avnet

**Affiliations:** 1 Orthopaedic Pathophysiology and Regenerative Medicine Unit, Istituto Ortopedico Rizzoli IRCCS, 40136 Bologna, Italy; nicola.baldini@ior.it; 2 Department of Biomedical and Neuromotor Sciences, University of Bologna, 401223 Bologna, Italy

**Keywords:** insulin receptor, acidosis, glucose metabolism, ion/proton transporters, acid-sensing ion channels

## Abstract

Most pathological conditions that cause local or systemic acidosis by overcoming the buffering activities of body fluids overlap with those diseases that are characterized by glucose metabolic disorders, including diabetes mellitus, inflammation, and cancer. This simple observation suggests the existence of a strong relationship between acidosis and insulin metabolism or insulin receptor signaling. In this review, we summarized the current knowledge on the activity of insulin on the induction of acidosis and, vice versa, on the effects of changes of extracellular and intracellular pH on insulin resistance. Insulin influences acidosis by promoting glycolysis. Although with an unclear mechanism, the lowering of pH, in turn, inhibits insulin sensitivity or activity. In addition to ketoacidosis that is frequently associated with diabetes, other important and more complex factors are involved in this delicate feedback mechanism. Among these, in this review we discussed the acid-mediated inhibiting effects on insulin binding affinity to its receptor, on glycolysis, on the recycling of glucose transporters, and on insulin secretion via transforming growth factor β (TGF-β) activity by pancreatic β-cells. Finally, we revised current data available on the mutual interaction between insulin signaling and the activity of ion/proton transporters and pH sensors, and on how acidosis may enhance insulin resistance through the Nuclear Factor kappa B (NF-κB) inflammatory pathway.

## 1. Introduction

Acid–base homeostasis and pH regulation are critical for both normal physiology and cell function, and is subjected to strong regulation. Precise maintenance of pH is needed, both at the extracellular level and in the cytosol, since changes in the extracellular space immediately impact on the intracellular cytosolic pH, and the cell machinery is very sensitive to changes in intracellular H^+^ concentration. To maintain the acid–base balance, extracellular pH is usually kept around neutral values and within narrow limits. The most important examples are arterial blood pH that is maintained between 7.36 and 7.44, or the pH of the venous blood that is around 7.6. As an immediate mechanism of defense against pH changes, several intracellular and extracellular buffers are available at different body districts. In the blood, the most important is the HCO_3_^−^/CO_2_ buffer system, but hemoglobin or plasma proteins also substantially contribute to the buffering activity with their numerous histidine residues. In addition, other proteins with histidine residues at other body districts, or citrate and hydroxyapatite in bone, have strong alkalinizing activity. Notably, interstitial fluids contain little pH buffering molecules and, at this level, the pH is likely to be lower and subjected to a more complex and dynamic gradient. In this case, the pH value varies strongly depending on the type of specific tissue, on the individual cell metabolic activity, and on the distance of the specific cell from the nearest capillary vessel. Due to technical difficulties, this has not been a well-investigated area. However, next-generation techniques allow for the real time measurement of live-cell metabolism, intracellular pH, and proton pumping, both through imaging instrumentations and biochemical assays, thereby opening up the possibility for a better comprehension of the phenomenon for the future.

Different pathological conditions may cause an overcoming of the buffering activities in bodily fluids. A multitude of potential causes of systemic and local acidosis exists. Local acidification may result from growth factors or cytokine stimulation of cell metabolism, vascular disease, ischemia, infection, tumors, or inflammation [1,2,3,4]. In fracture healing, immediately after the initial trauma, fracture hematoma is characterized by hypoxia and low pH [5]. Furthermore, as demonstrated by Marunaka et al., even in pre-disease stages, pH is drastically lowered in interstitial fluids around various tissues, including the brain [6]. At a systemic level, in addition to renal and respiratory diseases, a source of excessive accumulation of H^+^ are anaerobic exercise, gastroenteritis, the excessive consumption of proteins or of other acidifying substances, anemias, acquired immunodeficiency syndrome (AIDS), aging, and menopause, and last but not least, diabetes. Remarkably, most of the aforementioned situations overlap with those which occur with an altered glucose or insulin metabolism, suggesting a strong relationship between acidosis and insulin metabolism or insulin receptor signaling.

Insulin is the most potent physiological anabolic known agent, promoting the storage and synthesis of lipids, protein, and carbohydrates, and inhibiting their breakdown and release into the body’s circulation [7]. Insulin is particularly active in muscle and liver cells and is also often coupled with fatty acid uptake, especially in adipose cells [8]. However, all mammalian cells virtually possess insulin receptors (IR), and hence respond to insulin. Additional notable insulin target cells with a metabolic function include macrophages, endothelial cells, and insulin-producing pancreatic β-cells [9].

In this review, we described how insulin activity modulates pH regulation, and vice versa, how changes in pH—both at the extracellular or the intracellular compartments—regulate insulin signaling and glucose metabolism.

## 2. Insulin-Mediated Increase of Glycolysis and Local Acidosis

One of the major functions of insulin is a stimulatory effect on glycolysis that occurs when there is a rise in the level of circulating glucose. In turn, glycolysis causes lactic acid production. Hyperlactatemia is a recurrent clinical feature of diabetic patients [10] and has been directly associated with acidosis through a cause-and-effect relationship. However, the correlation between the high blood concentration of lactate and acidosis in diabetes is far from being clear. In a clinical context, lactic acidosis can occur either due to excessive production of lactate at the tissue level or impaired lactate metabolism. Notably, hyperlactatemia may also occur with a normal serum pH or with alkalosis due to the presence of a mixed acid–base disturbance, where a concomitant respiratory or metabolic alkalosis masks the effect of the elevated lactic acid concentrations.

**Figure 1:**
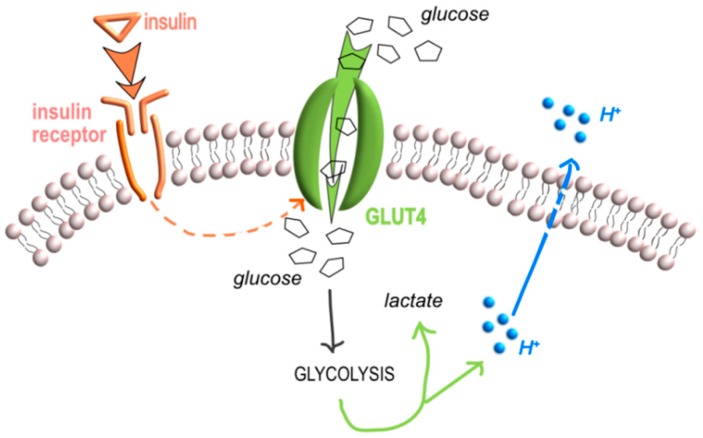
Insulin stimulation produces extracellular acidification. The insulin stimulated glucose uptake from the glucose transporters (GLUT) and glycolysis. Accumulation of lactate from glycolysis leads to a progressive increment of lactate and proton concentration. Protons are then transported across the cell membrane through several transporters, including monocarboxylate transporters (MCT), that also transport lactate to the extracellular space (orange arrow, the route of insulin; green allows, the route of the products of glycolysis, lactate and protons; blue arrow, the route of protons).

In more detail, regarding the excessive production of lactate, it is important to note that it has often been mistakenly thought that acidosis associated with lactic acid production is due to the dissociation of lactic acid and formation of H^+^ [11]. Already in 2004, Robergs disproved this conception by showing that the reaction catalyzed by lactic dehydrogenase (LDH) produces lactate and not lactic acid, and that production of lactate rather consumes H^+^ [12]. Based on the same concept, more recently, Corbet et al. discussed that lactate and H^+^ ions are separate entities [13]. In particular, under deep hypoxia, lactate and H^+^ are produced independently and only limited fractions of each combine to form lactic acid, as the pKa (~3.9) for the carboxyl hydrogen dissociation remains well below cytosolic pH and even extracellular pH. Furthermore, when most glucose-derived pyruvate is reduced into lactate, the full conversion of glucose into two lactate molecules and two H^+^ ions in hypoxic cancer cells is only theoretical since some glucose molecules are diverted into other metabolic pathways [13]. However, the lactate gradient across the cell membrane influences H^+^ efflux since lactate is transported across the cell membrane by monocarboxylic acid transporters (MCT), mainly by the MCT4, through a symport of H^+^ across the cell membrane [14]. Finally, once lactate reaches the extracellular space, the lactate-based acid–base balance is influenced by several interacting variables—not only including the electrical charge balance and conservation of mass, but also the equilibria among bicarbonate ion formation, carbonate ion formation, water dissociation, and weak acid dissociation. Thus, in summary, accumulation of lactate might result in an increase of extracellular acidification even if it strongly depends on different variables (Figure 1).

In addition, several proton transporters other than MCT can be used to promptly carry protons outside the cell. For more details, see different reviews available in this specific field [4,15].

Regarding the impaired lactate metabolism and its role in hyperlactatemia-induced acidosis, lactate can by uptaken to be metabolized by various cells and tissues, such as liver, germ cells, and neurons, converting to pyruvate via LDH and subsequently serving as an energy source. In this case, lactate is uptaken mainly by the MCT1 isoform, thereby causing a final alkalinization effect [16]. Thus, a reduced lactate metabolism may indirectly cause acidification.

Finally, it should be considered that lactate exists as two optical isomers in nature: d-lactate and l-lactate [17]. l-lactate is by far the more abundant form in humans and mammals, existing at concentrations 100 times greater than d-lactate in blood. d-lactate can be metabolized by human cells, although at a much lower level than l-lactate since it is mainly metabolized by the bacteria of the gut microbiota. To date, it is not very clear whether the hyperlactatemia in diabetic patients is due to the increased concentration of l-lactate and d-lactate, and whether lactate acidosis is mainly due to the high concentration of d-lactate in the blood, rather than to l-lactate [18,19].

Going back to the association between glycolysis and acidosis, based on Warburg’s findings [20], direct evidence of a correlation between glycolysis and extracellular acidosis in cancer has been produced by combining magnetic resonance imaging, through coupling ^18^F-fluoro-2-deoxy-d-glucose ([^18^F]FDG) positron emission tomography (PET) and acido chemical exchange saturation transfer magnetic resonance imaging (acidoCEST MRI) [21,22]. These studies have demonstrated a significant inverse correlation between extracellular tumor pH and [^18^F]FDG uptake.

In conclusion, at least at the cell level, insulin binding to IR and the following activation of glycolysis might be a direct cause of interstitial acidification.

## 3. Acidosis-Mediated Effects on Insulin Activity and Resistance

### 3.1. Effects of Acidosis on Insulin Sensitivity and Release

pH is known to modify insulin activity, and that acidification is considered as a mechanism of insulin resistance. By using in vivo models, it was demonstrated that pH regulation modifies basal and glucose-stimulated insulin secretion since hyperglycemia induced by the glucose load decreases more rapidly in alkalotic than in normal rats [23,24]. Previously, metabolic ketoacidosis was considered as the main cause. This was due to the fact that, both in human diabetic patients with metabolic acidosis and in animal models of diabetes, insulin resistance and the lack of the induction of glucose uptake and glycolysis were accompanied by increased concentrations of ketone bodies in the blood flow [25], and because animal models of severe ketoacidosis develop insulin resistance [26]. In the late nineties, studies on human patients on hemodialysis showing insulin resistance demonstrated that alkalinizing treatment with oral sodium bicarbonate (NaHCO_3_) significantly increased insulin sensitivity and secretion [27]. Finally, by using the euglycemic clamp technique, Reaich et al. further confirmed that NaHCO_3_ treatment of patients with metabolic acidosis from chronic renal failure significantly increased insulin sensitivity [28].

**Figure 2:**
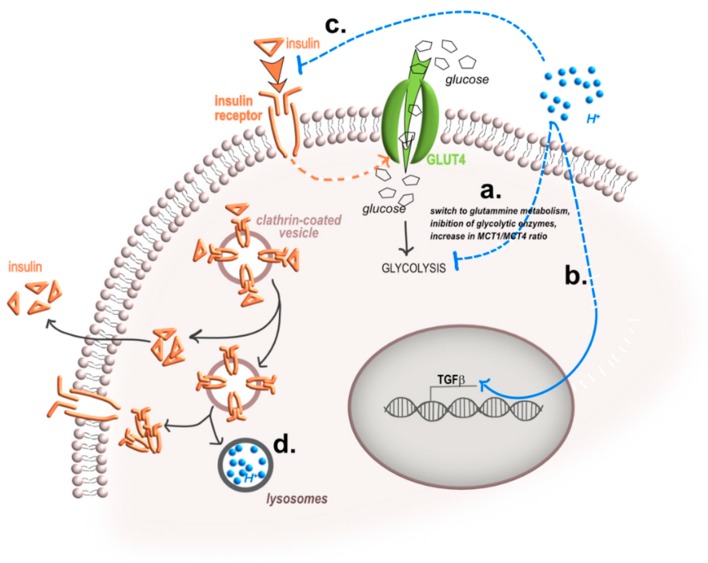
Effects of acidosis on insulin sensitivity and release. Low extracellular pH may interfere with insulin sensitivity and release in different ways. (**a**) Acidosis switches the cellular metabolism from glycolysis to other metabolic pathways, like glutamine metabolism via the Sirtuin 1 (SIRT-1) deacetylase activity that deacetylates hypoxia-inducible factor (HIF) proteins, or via the inhibition of the expression of glycolytic enzymes or through the increase of monocarboxylate transporter MCT1/MCT4 pathway; (**b**) acidosis induces the transcription and the protein expression of TGF-β that, in turn, inhibits insulin release at the systemic level; (**c**) acidosis reduces at least 40% the binding affinity between insulin and IR; (**d**) acidosis interferes with the recycling process of both IR and insulin, by modifying the lysosome intraluminal pH and the number of lysosomes (blue arrow, the route of protons, black arrows, the route of insulin/IR complexes; T-bar, inhibition).

The cause-and-effect relationship between changes in pH environments and alterations in insulin activity is still piecing. This is possibly due to the several variables that make the picture increasingly more complex. One possible cause of acidosis-induced insulin resistance has been demonstrated in transformed cells. Exposure to low pH directly reduces glycolysis, both after acute [29,30] or chronic exposure [31]. In the latter case, glycolysis reduction is obtained through a switch to glutamine metabolism and is mediated by a nicotinammide adenina dinucleotide (NAD)(+)-dependent increase in sirtuin 1 (SIRT-1) deacetylase activity that deacetylates hypoxia-inducible factor (HIF) proteins to adapt to the acidic environment [31] (Figure 2a).

In addition, as demonstrated in human breast cancer Bcap37 cells, an acid pH may decrease glucose uptake and the glycolytic flux by directly inhibiting the activities of most glycolytic enzymes, including hexokinase (HK), aldolase, pyruvate kinase (PK), and LDH, with particular reference to phosphoglucose isomerase (PGI), phosphofructokinase (PFK), triosephosphate isomerase (TPI), and enolase, and through a mechanism that is independent of the presence of lactic acid [32]. Furthermore, incubation with an acid medium may down-regulate glycolysis also in normal cells. As an example, in mesenchymal stromal cells (MSC), exposure to an acid medium (6.6–6.7) changes the cellular metabolic profile since the expression of glucose transporters is significantly reduced and the ratio between MCT1 and MCT4 is up-regulated [33]. MCT1 mediates lactate uptake. Conversely, MCT4 facilitates the release of lactate into the extracellular environment, and both MCT1 and MCT4 are expressed both from normal and cancer cells [15,34,35]. In acid-loaded MSC, high levels of MCT1 increase lactate upload, whereas inhibition of GLUT1 and GLUT3 reduces glucose uptake, implying that under this stress condition MSC switch from glycolysis to OxPhos. This metabolic switch is also confirmed by a reduced expression of lactate dehydrogenase A (LDH-A) and pyruvate kinase isozymes m2 (PKM2) [33]. According to these data, it is possible to speculate that, at the individual cell level, cytosolic pH controls the glycolytic rate through a feedback mechanism because of the need to reduce the production of protons from glycolysis and thereby indirectly causing insulin resistance.

Finally, at a systemic level, it is known that acidification of the extracellular fluids stimulates the release of TGF-β in normal cells, since either in MSC, in proximal tubular cells, or in hippocampal cultures, in comparison to normal conditions, acidosis causes a fourfold increase of the level of expression of TGF-β or of TGF-β1 bioactivity [33,36,37] (Figure 2b). In turn, the systemic release of TGF-β modulates pancreatic development and islet homeostasis and function as TGF-β inhibits the replication of β-cells [38,39,40]. These data indirectly suggest that the acid-induced systemic release of TGF-β might decrease the circulating level of insulin, thereby partially contributing to the feedback inhibitory mechanism of acidosis that ultimately results in insulin resistance.

### 3.2. Effects of Acidosis on Insulin Receptor Expression, Activation, and Signaling

Acid-induced insulin resistance has been observed in several cell types [25,41,42,43]. A possible very simple reason for this effect is reduced insulin binding to its receptor at high proton concentrations (Figure 2c). Igarashi et al. have elegantly shown that in adipocytes, lowering of extracellular pH is associated with a reduced insulin binding rate (up to 70%) [25]. In this experimental set, the authors demonstrated that such an effect was mainly due to a reduction in insulin binding affinity to its binding sites, without affecting the number of IR per cell. A reduced insulin binding rate at low pH, compared to neutral pH, has also been demonstrated in fibroblasts, erythrocytes, and myoblasts [41,42,43,44,45,46]. In particular, in L6 myoblasts, the insulin binding to IR was down-regulated up to 50% at pH 6.8 [42]. Acidosis thus has the potential to directly affect the insulin-stimulated glucose uptake by interfering with the first step of the insulin signaling pathway, the IR activation, which, as a consequence, might impair the downstream IR signaling. Insulin stimulates glucose uptake mainly in a phosphatidylinositol 3-kinase (PI3K)-dependent pathway. After binding to IR, the autophosphorylation of IR and of the second messenger insulin receptor substrate-1 (IRS-1) leads to the activation of PI3K and the downstream Akt-mediated signaling. As a confirmation of this hypothesis, in rat myoblasts, a low pH (6.8) significantly affected the 100 nM insulin stimulation of Tyr1146, Ser473, and Thr308 phosphorylation of IR. On the contrary, the expression of IR was not affected [42]. By using radiolabeled insulin, the same authors also demonstrated that the reduced IR phosphorylation was due to decreased insulin binding affinity to the receptor that was inversely correlated to the level of pH [42]. Notably, extracellular acidosis might also directly interfere with MEK/ERK (mitogen-activated protein kinases/extracellular signal-regulated kinases) signaling and Akt activation, regardless of insulin stimulation [4].

Finally, the level of expression of IR at the plasma membrane is strongly dependent on receptor recycling and lysosomal activity. Receptor-mediated endocytosis is an essential mechanism for several important physiological processes, and similarly to other receptor tyrosine kinases, IR undergoes ligand-induced internalization. After internalization, IR can be recycled to the cell surface, or can also undergo lysosomal degradation [9]. This event requires a specific amino-acid motif located at the intracellular part of the IR juxtamembrane domain. This motif targets the receptor to clathrin-coated pits, and from there to lysosomes, where the level of acid pH is thought to enhance insulin release [47]. In many cell types, insulin itself stimulates receptor endocytosis and accelerates the rate of receptor degradation. This process, called down-regulation, results in a decrease in the number of cell surface receptors and may be important to reduce signals initiated by insulin binding [48]. Whether this is a mechanism of insulin resistance is still controversial. However, it is proven than continuous exposure to high insulin levels induces subtle derangements of intracellular receptor trafficking and insulin degradation, and these alterations may contribute to insulin resistance of hyperinsulinemic states, such as obesity and Type 2 diabetes [49]. The lysosomal activity that regulates the internal trafficking of IR, as well as of insulin, might be affected by a reduced extracellular pH value. As an example, in breast cancer cells, rhabdomyosarcoma cells, and osteosarcoma cells, incubation with an acid medium increased the lysosomal diameter and decreased the luminal pH [50,51,52]. Thus, changes in the extracellular pH that are accompanied by changes in the intracellular pH and in lysosomal pH might be a critical factor for insulin secretion and insulin resistance (Figure 2d).

### 3.3. Effect of Acidosis on the Expression of GLUT Transporters

Insulin increases energy storage or utilization mainly through the regulation of the uptake of glucose by inducing the expression on the cell membrane of the GLUT4 transporter [53]. In patients with type 2 diabetes, the expression levels of GLUT4 in skeletal muscles are significantly decreased, resulting in a decreased glucose-processing ability. More rarely, GLUT1 has also been associated with insulin-mediated glycolysis regulation [54]. In more detail, insulin-induced glucose uptake is heavily regulated by the translocation of GLUT transporters to the cell membrane and the lasting of GLUT4 expression at this site. As for the IR, GLUT4 is continuously recycled and relocated between the plasma membrane and the intracellular compartment [53]. GLUT4 recycling is the result of consecutive and tightly regulated steps, including endocytosis, sorting into specialized vesicles, exocytosis, tethering, and fusion of the protein. In the absence of insulin, GLUT4 slowly recycles between the plasma membrane and vesicular compartments within the cell, where most of GLUT4 resides. Conversely, insulin stimulates the translocation of a pool of GLUT4 to the plasma membrane through a process of target endocytosis, where GLUT4 endocytosis is simultaneously attenuated [7]. The whole phenomenon involves signal transduction from the IR, vesicle trafficking (sorting and fusion processes), and actin cytoskeleton modifications, which are all supposed to require small guanosintrifosfato hydrolases (GTPases). In particular, various members of the Ras, Rad, Rho, Arf, and Rab families regulate the traffic of the GLUT4-containing vesicles [55], like Rab4b in adipocytes [56]. Also, Rab11 has been associated with GLUT4-containing vesicles’ redistribution in response to insulin [57,58]. As discussed for IR, changes of extracellular pH might regulate the activity of endosomal vesicles, thereby determining the extent of GLUT4 re-localization and recycling. Actually, in HSG cells, extracellular acidosis up-regulated Rab11b expression and protein abundance and caused, as an example, the translocation of the vacuolar proton pump V-ATPase from intracellular pools toward the plasma membrane [59]. To date, it is not known how lowering the pH modulates small GTPases expression and their activity in determining GLUT recycling. It is demonstrated, however, at least in MSC, that the incubation with an acid medium promotes the down-regulation of the expression of GLUT1 and GLUT3 [33]. However, this hypothesis deserves further investigation.

## 4. Intracellular pH Regulation by Proton/Ion Transporters and Insulin Secretion

To avoid the death-inducing stimulus of a high intracellular accumulation of protons that can be derived both from a reduction of extracellular pH or an increase of glycolysis, cells express different proton flux regulators at different extents, depending on the tissue of origin [15]. These pump/transport extrude protons out of the cytosol to maintain the cytosolic pH. Among these, V-ATPase is widely expressed at the lysosomal membrane, whereas Na^+^/H^+^ exchangers (NHE), MCT, and carbonic anhydrases (CA) are expressed at the cytoplasmic membrane [60,61,62]. The activity of these transporters/pumps have the net effect to reduce the luminal lysosomal pH and the pH of the extracellular space [4,15]. As these transporters are activated, intracellular pH (pHi) rises, albeit temporarily. However, chronic exposure to an excess of protons produces a paradoxical gradual and sustained increase of pHi. One of the most common examples for the constant rise of pHi in concert with chronic extracellular acidosis is cancer [63].

The rise of pHi is widely known as a critical factor for insulin secretion. One of the most studied models in this context are pancreatic β-cells. Glucose load in these cells, at concentrations above 3–5 mM, induces cellular depolarization and pHi transitory increments (up to 6.4–6.8 values) that directly stimulates insulin secretion [64,65,66]. Furthermore, Nabe et al. have demonstrated that diphenylhydantoin, a drug used for the treatment of epileptic patients, inhibits glucose-induced insulin release from pancreatic islets by decreasing intracellular proton concentration [67].

**Figure 3:**
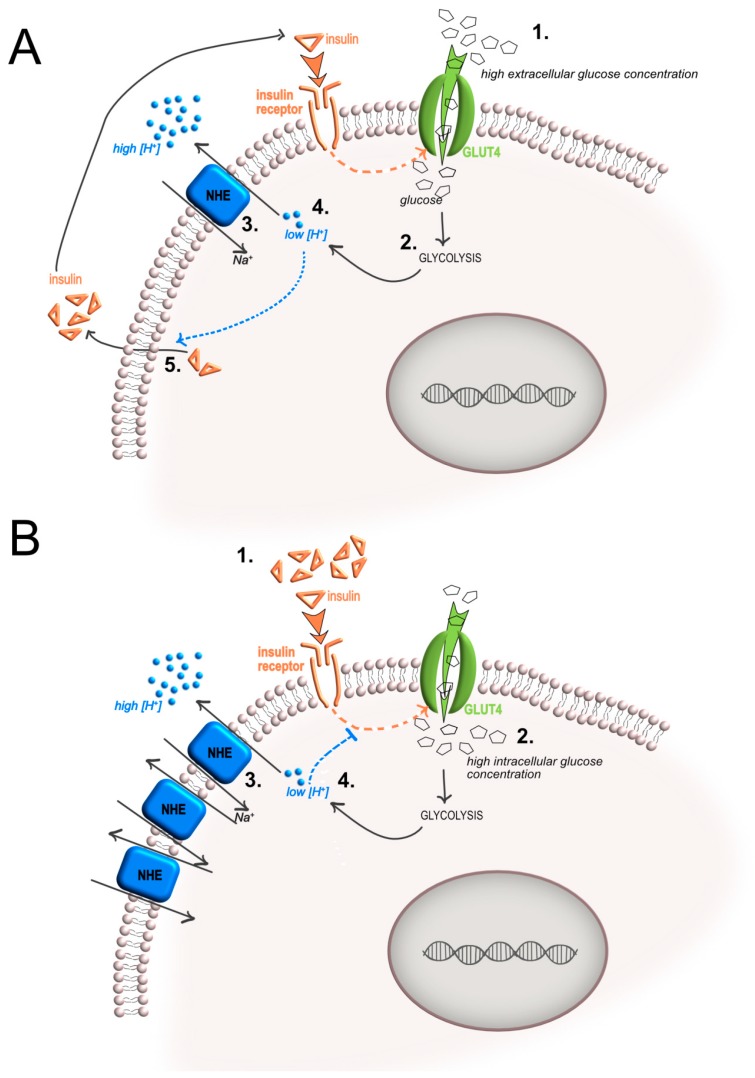
Intracellular pH regulation by NHE transporter, and insulin secretion and sensitivity. (**A**) A rise in intracellular pH (pHi) after glucose uptake is due to the activity of the NHE Na^+^/H^+^ antiporter that is counteracting the glycolysis-induced intracellular acidification. The increase of pHi then directly stimulates insulin release; (**B**) in chronic hyperglycemia, a negative feedback mechanism may prevent supplementary uptake of glucose that, after chronic exposure to insulin, would excessively acidify the cytosol. This mechanism might cause a paradoxical effect mediated by a high level of NHE expression, thereby reducing insulin sensitivity (orange arrow, the route of insulin; blue arrow, the route of protons; T-bar, inhibition).

Although such a mechanism has not been fully elucidated, the increase of pHi after glucose loading in β-cells seems to be based on the presence of extracellular Na^+^ and on the activity of sodium–hydrogen exchangers (NHE), since it is inhibited by 5-(*N*-ethyl-*N*-isopropyl) amiloride, an NHE inhibitor [65,66]. According to these data, it is reasonable to speculate that the rise of pHi after glucose uptake is due to the activity of the NHE Na^+^/H^+^ antiporter that counteracts the glycolysis-induced intracellular acidification (Figure 3A).

A similar phenomenon has also been observed in muscle cells. At 37 °C, acute exposure to insulin (10^−7^ M) and the resulting increase in glucose uptake produced an increase of pHi of 0.11 units in 10 min. This increase became apparent 2 min after the addition of the hormone, and maximal elevation of pHi was observed after 10 min, remaining elevated for up to 60 min [68]. In this case, pHi alkalinization was also prevented by amiloride. However, a recent review that summarized the most recent results on NHE and insulin clarified that plasmalemmal full-length NHE1 defends β-cells from intracellular acidification, but has no role in stimulus-secretion coupling and is not causally involved in glucose-induced alkalinization of the β-cell [69]. The discrepancy of the data reported in the literature so far on the relationship between the alkalinization activity of NHE and the stimulatory effect of a pHi rise on insulin secretion suggests the need to better clarify the phenomenon.

NHE also contributes to pHi regulation in arterial endothelial and smooth muscle cells and might thereby strongly influence blood pressure. In this respect, it is interesting to note that hypertensive patients often develop insulin resistance and hyperinsulinemia [70]. This is a chronic condition that may cause a paradoxical opposite effect in respect to those represented in Figure 3A. By using human red blood cells from patients with essential hypertension compared to red blood cells from normotensive subjects, it appeared that hypertensive patients had elevated basal NHE activity but a blunted response to insulin [71]. In this setting, a negative feedback mechanism might be suggested to prevent supplementary uptake of glucose that, after chronic exposure to insulin, would excessively acidify the cytosol (Figure 3B).

As for NHE, CA activity and its regulation of pHi might be involved in the altered metabolism of chronic hypertensive patients. CA is a well-characterized, pH regulatory, zinc-containing enzyme in most of the tissues in the body that catalyzes the reversible hydration of carbon dioxide: CO_2_+ H_2_O<-->HCO_3_^−^+H^+^. Changes in CA activities have already been associated with cancer-altered metabolism [15]. By using p-nitrophenyl acetate as a substrate and acetazolamide, a CA9-specific inhibitor, in erythrocytes from normotensive and essential hypertensive subjects, Parui et al. demonstrated that two different levels of CA activities could be detected in these patients. One group showed much lower CA activity (mean ± SD, 0.88 ± 0.19 U/min/mL), whereas the other group showed higher CA activity (mean ± SD, 1.77 ± 0.23 U/min/mL) than normotensive controls (mean ± 1 SD, 1.41 ± 0.1 U/min/mL), and concluded that essential hypertensive patients were associated with altered CA activity [72]. Although the role of CA activity and other transporters that might modulate pHi in insulin sensitivity remains largely unexplained, these data altogether pave for pharmacological manipulations of pHi to treat insulin resistance.

## 5. Insulin Receptors and Sensing Extracellular Acidosis

Insulin-stimulated glucose uptake and glycolysis are reduced at low extracellular pH, whereas insulin secretion is enhanced when pHi rises. It is then possible to speculate that specific mechanisms of sensing pH variation and gradient between the extracellular and the intracellular spaces exist, accounting for the activation of the insulin pathway and metabolic activity. Several extracellular proton-sensing mechanisms are expressed on cell membranes in many biological systems. The existence and the apparent redundancy of multiple pH surveillance systems attest to the concept that acid–base regulation is vital for cell and tissue homeostasis [73]. Indeed, in addition to carrying the multiple currents evoked by protons, these acid-sensing ion channels play fundamental roles in cell signaling and allow the host cell to respond to benign or harmful environmental changes [74,75]. There are at least two types of proton-sensing mechanisms, which sense variations of the extracellular proton concentration and transduce their signal inside the cells: acid-sensing ion channels (ASICS) and transient receptor potential vanilloid (TRPV) ion channels. ASICS and TRPV can detect a broad range of physiological pH changes, especially during pathological and synaptic cellular activities in neurons. Emerging evidence indicates that many of the acid-sensitive ion channels and receptors also play a role in the acid-evoked feedback regulation of homeostatic reactions, and can be expressed from cells other than neurons. As an example, ASICs have been detected in cells of the mesenchymal lineage [76], and with particular regard to ASIC3, knockout mice are protected against age-dependent glucose tolerance with enhanced insulin sensitivity [77]. By using the TRPV inhibitor capsaicin or TRPV knock-out mice, several types of TRPV have been revealed to contribute to cell function in pancreatic β-cells, including glucose metabolism and insulin secretion [78].

In addition to ionotropic ion channels, metabotropic proton-sensing G protein-coupled receptors (GPCR) have been recently identified as a proton-sensing machinery. While ionotropic ion channels usually sense strong acidic pH, proton-sensing GPCRs sense a pH of 7.6 to 6.0 and mediate a variety of biological actions under neutral and mildly acidic pH environments [79]. Referring to ovarian cancer, in addition to ASIC3, the proton-sensing GPCR G protein-coupled receptor 1 (OGR1) has been proven to function in physiological events, such as insulin secretion and/or normal glucose metabolism. OGR1 is activated by neutral or mildly acidic pH (pH 7.4~7.0) and stimulates the processes of the KATP channel and/or VDCC, and thereby enhances glucose-stimulated insulin secretion through the phospholipase C (PLC)/Ca^2+^ signaling pathways [79]. Finally, in normal pancreatic β-cells, OGR1 agonists (small molecule 3,5-disubstituted isoxazoles) stimulate insulin synthesis [79].

In conclusion, current fragmentary data suggest that a fine-tuning of insulin activity and sensitivity is under the control of pH surveillance systems, a concept that might strongly impact on our understanding of the regulation of glucose homeostasis. However, the role of proton-sensing receptors in insulin sensitivity and release is still unclear and deserves further investigation.

## 6. Insulin Receptor-Induced Inflammatory Pathway and Acidosis

Tissue acidification (pH decrease of at least 0.5–1.0 pH unit) is commonly associated with inflammation [80]. Indeed, after sensing the extracellular pH changes, ASICs might initiate an inflammatory response [75]. As an example, lowering pH promotes the expression of the mRNA messenger of IL-6, and IL-8, as well as of NF-κB1, RelA, and RelB already after 3 h from the exposure to acidosis (pH 6.8), and after 24 h, it promotes the intranuclear protein expression of members of the NF-κB family and the release of the inflammatory cytokines IL-6 and IL-8, both in human MSC and osteoblasts [76,81]. Similarly, GPCRs that can sense the acid stimulus can mediate inflammatory and related responses, like for T-cell death-associated gene 8 (TDAG8, also known as GPR65), that enhances the production of IL-6, TNF-α, and IL-1β in the infarcted hearts of mouse models [82].

Activation of inflammatory pathways by acidosis can, in turn, impair glucose metabolism and cause systemic insulin resistance. Lipid-induced insulin resistance has been associated with inflammation in epidemiological studies, and by means of different models, several authors have demonstrated that induction of NF-κB activation reduces net insulin-stimulated glucose uptake. For an extensive review, see Reference [83]. In particular, Chang et al. have shown that in C2C12 myotubes, transfection with siRNA against the NF-κB member, the nuclear factor of kappa light polypeptide gene enhancer in B-cells inhibitor alpha insulin receptor substrate 1 (IκBα), for 24 h leads to a twofold induction of NF-κB activation, reduced net insulin-stimulated glucose uptake by 30%, GLUT4 translocation by 35%, Akt phosphorylation by 31%, and a 0.7-fold increase in insulin-stimulated insulin receptor substrate 1 (IRS-1) phosphorylation [84]. The enhancement of GLUT4 expression via NF-κB has also been confirmed in other models [85]. Furthermore, other authors have demonstrated in adipocytes that the IκB kinase β (IKKβ), c-Jun N-terminal kinase (JNK), and S6K and mTOR32 kinases carry out inhibitory phosphorylation of IRS-1, thus causing insulin resistance [9]. Both experimental and clinical studies now converge to show that several ILs contribute to the pathology and physiology of Type 2 diabetes through their interaction with insulin signaling pathways and β-cell functions. Notably, IL-1, which is a major proinflammatory cytokine, is present at increased levels in patients with diabetes mellitus, and could promote β-cell destruction and alter insulin sensitivity [86]. Likewise, IL-6 has been suggested to be involved in the development of obesity-related and diabetes mellitus Type 2-related insulin resistance. The action of IL-6 on glucose homeostasis is also complex, and integrates central and peripheral mechanisms [87]. Overall, these data demonstrate that acidosis has the potential to indirectly cause insulin resistance via the activation of NF-κB pathways and the release of inflammatory cytokines, an already well-known cause of chronic inflammation in diabetic patients.

## 7. Conclusions

Acidosis modulates insulin sensitivity and resistance in many different and complex ways. Notably, the timing of exposure to acidosis is a crucial factor in this context, since acute and chronic effects of acidosis may have completely opposite effects. To control acid-related diseases, several approaches have been suggested in mouse models, including the use of bicarbonate [4,88]. Once translated to the clinical practice, however, results obtained from these studies might produce quite uncertain and dangerous effects in humans. For diseases associated with insulin resistance, like for type 2 diabetes, decreasing dietary acid load may be an achievable alternative and a relevant route to improve glucose homeostasis and prevention on a long-term basis. However, limitations related to patient acid load estimation, nutritional determinants, and metabolic status considerably flaws available findings, and the lack of solid data on the background pathophysiology contributes to the questionability of results. Furthermore, evidence from interventional studies is very limited and the trials carried out so far report no beneficial results following alkali supplementation [89]. Nevertheless, the relation between insulin metabolism and the regulation of pH is an exciting and fascinating field of investigation for the identification of novel therapeutic approaches. Furthermore, in clinical practice, data that have been collected so far suggest considering pH not only as a body parameter to be continuously kept under control to avoid the dangerous effects of systemic acidosis or alkalosis, but also as a main driver that directly modulates crucial biological processes, either at the systemic, cellular, or molecular level.

## Abbreviations

acidoCEST MRI	acido chemical exchange saturation transfer magnetic resonance imaging
AIDS	acquired immunodeficiency syndrome
ASICS	acid-sensing ion channels
CA	carbonic anhydrases
[^18^F]FDG PET	^18^F-fluoro-2-deoxy-d-glucose positron emission tomography
GLUT	glucose transporters
GPCR	metabotropic proton-sensing G protein-coupled receptors
GTPases	guanosintrifosfato hydrolases
HE	Na^+^/H^+^ exchanger
HIF	hypoxia-inducible factor
HK	hexokinase
IκBα	the nuclear factor of kappa light polypeptide gene enhancer in B-cells inhibitor alpha
IR	insulin receptor
IRS-1	insulin receptor substrate 1
LDH	lactic dehydrogenase
LDH-A	lactate dehydrogenase a
MCT	monocarboxylate transporter
MEK/ERK	mitogen-activated protein kinases/extracellular signal-regulated kinases
MSC	mesechymal stromal cells
NAD	nicotinammide adenina dinucleotide
NF-κB	Nuclear Factor kappaB
NHE	sodium–hydrogen exchanger
NSIS	nutrient-stimulated insulin secretion
OGR1	ovarian cancer G protein-coupled receptor 1
OxPhos	oxidative phosphorylation
PFK	phosphofructokinase
PGI	phosphoglucose isomerase
pHi	intracellular pH
PK	pyruvate kinase
PKM2	pyruvate kinase isozymes m2
SIRT-1	Sirtuin 1
TGF-β	transforming growth factor beta
TPI	triosephosphate isomerase
TRPV	transient receptor potential vanilloid
V-ATPase	vacuolar ATP-ase
